# Ethnic Variations in Healthcare Service Utilization and Access in Vietnamese Mountainous Setting

**DOI:** 10.1155/2021/6650303

**Published:** 2021-04-22

**Authors:** Tuan Le Anh, Ha Vu Ngoc, Dua Nguyen Nhu, Anh Truong Thi Mai, Tam Ngo Thi, Bach Nguyen Xuan, Kien Pham Trung, Hung Hoang Nam, Anh Nguyen Thi Hoang, Thanh Luong Trung

**Affiliations:** ^1^VNU University of Medicine and Pharmacy, Vietnam National University, Hanoi 100000, Vietnam; ^2^Faculty of Health Sciences, Thang Long University, Hanoi 100000, Vietnam; ^3^Hospital of Vietnam National University, Hanoi 100000, Vietnam

## Abstract

Evidence of health service use and access across different target groups is essential for policy development, health promotion, and promotion of equity in healthcare. This study aims to look at ethnic variations in health service use and access among residents in mountainous areas of Vietnam. A cross-sectional descriptive study was conducted on 321 adults from two mountainous communes in Bac Kan province. Healthcare service use and access were evaluated by using a structured questionnaire. Zero-inflated Poisson regression was used to examine the ethnic variations in the healthcare service use and access. Of 321 mountainous residents, 63.6% used health services in the previous 12 months, of which 24.9% respondents used inpatient services and 47.9% used outpatient services. The number of outpatient medical services used by the Tay participant was higher than that of the Kinh and other ethnic groups (*p* < 0.05). Multivariate regression results showed that compared to Kinh people, Tay people had a higher number of outpatient service use (Coef. = 0.25, *p*=0.04), while people in other ethnicities had a lower number of service use (Coef. = −0.64, *p*=0.01). Meanwhile, no difference was found among groups regarding the number of inpatient service use (*p* > 0.05). This study showed the ethnic differences in outpatient use of health services among communities living in the northern mountainous setting of Vietnam.

## 1. Introduction

Health service access and use are critical indicators to help assess the effectiveness of the health system in delivering healthcare [1–3]. Inequality in access to health services can significantly affect the health status and quality of life of people in inaccessible places [4, 5]. Understanding and having evidence regarding health service access and use can allow policymakers to identify vulnerable populations that need support, thereby providing appropriate solutions to improve accessibility and health in these groups.

Vietnam has achieved some initial success in healthcare, thanks to the widespread coverage of the public health system, especially the primary healthcare, in district and commune levels [6]. By 2019, Vietnam had 700 district health centers and 11,083 commune health stations (with 49,544 beds) [7]. The availability of primary healthcare facilities in all localities is a significant effort of the Vietnamese health system to ensure that people have access to essential preventive, management, and treatment services. However, there is still inequality in health service access and use among people between urban and remote areas [1, 8]. People living in mountainous areas still face many problems related to an underdeveloped economy, limited access to education, and healthcare [9, 10]. A previous study suggested that people in remote and mountainous areas have a high rate of health problems, and there were many social barriers in accessing healthcare services, even with services provided at commune health stations [11].

Bac Kan is a northern mountainous province of Vietnam, inhabited by the Kinh, Dao, Thai, and many other ethnic minorities. The difficult terrain characteristics, as well as the complexity of the population structure, may be factors that influence the use and access to healthcare services. Ethnic minorities not only live in disadvantaged geographical positions but also their socioeconomic conditions are also deficient [12, 13]. Several social and health policies have been implemented to address this issue, such as the Government's 135 programs [14]. To date, there is a lack of evidence to consider the differences in healthcare access and use among different ethnic groups in this region. This study aims to look at ethnic variations in health service use and access among residents in mountainous areas of Vietnam.

## 2. Materials and Methods

### 2.1. Study Design and Setting

We conducted a cross-sectional study in Bac Kan province in 2017. Thanh Mai and Thanh Van communes of Cho Moi district were selected for the study. Participants were selected from the list of the local population using the systematic random sampling method. Village health workers supported the study by visiting households and inviting eligible people to visit commune health stations for study enrolment and a medical checkup. Selection criteria included (1) being a resident of the study area and (2) 18 years of age or older. Among 330 invitations, 321 residents (97.3%), including 172 people from Thanh Van commune and 149 people from Thanh Mai commune, agreed to participate in this study.

### 2.2. Variables and Measurement

Face-to-face interviews were conducted by investigators who were students of the University of Medicine and Pharmacy, Vietnam National University, Hanoi. All data collectors were trained to use structured questionnaires for data collection. The questionnaire included socioeconomic information, health status, and health service access and use.

Socioeconomic characteristics included age, gender (male/female), ethnicity (Kinh/Tay/others), education level (elementary/secondary/high school or above), marital status (single/having spouse/partner), and income. We also asked the smoking status (never/smoking/used to smoke) of the respondents. People were asked to report their acute symptoms in the last four weeks and chronic conditions in the last three months.

Health service access and use in the last 12 months were asked to be recalled, including having health service utilizations (yes/no), types of health service utilization (inpatient/outpatient), the number of inpatient/outpatient service use in the last 12 months, and health facilities they visited (provincial hospital, district hospital, commune health clinic, or other facilities). We also asked people to report the first facility they visited when they had an illness and the reasons for this selection. Moreover, distance from home to the commune health center and experiencing difficulty if going to the commune health center (yes/no) were also asked.

### 2.3. Statistical Methods

The data were entered using EpiData 3.1 and analyzed using Stata 14.0 software (Stata Corp LP, College Station, Texas, US). Chi-square test was used to examine categorical variables among different ethnic groups, while Kruskal–Wallis test was used to compare the median of continuous variables among these groups (because all continuous variables had nonnormal data). We used multivariate zero-inflated Poisson regression models to determine factors related to the number of inpatient or outpatient service use in the last 12 months. Potential independent variables included sociodemographic characteristics (age, gender, ethnicity, education level, marital status, smoking status, number of acute symptoms in the last four weeks and number of chronic conditions in the last three months, and distance from home to the commune health center). The data on the number of inpatient/outpatient service use in the last 12 months were the count data that had a number of zero counts, and the zero-inflated Poisson regression model is particularly useful for such data. This model is an appropriate technique for count data with excess zeros (e.g., none of the service use in the last 12 months) [15]. Its concept mentioned that the variable only has a zero value with probability *p* and Poisson random value with probability (1 *− p*). The model is formulated as follows:

(1) $$\begin{array}{c} \log\left(Y\right)=a+bX+e, \end{array}$$

where log (*Y*) is the log incidence of inpatient/outpatient service use

 
*a* and *b* are the parameters of the regression
  X represents the independent factors
 
*e* is the residual that could not be explained by the model

Akaike information criterion (AIC), Bayesian information criterion (BIC), and log likelihood were presented to illustrate goodness-of-fit indices. Statistical significance was detected with *p* < 0.05.

## 3. Results

Table 1 shows that the average age of participants was 51.7 ± 14.7 years. The Tay people were a major group with 71.7% (mean age = 52.0), following by the Kinh people (13.1%, mean age = 55.0). The majority of the samples was male (67.3%). There were 76.2% Kinh samples being male, while the proportion in Tay and other ethnics was 68.7% and 53.1% (*p*=0.044). Most of the samples had secondary education (47.5%), of which people in other ethnicities had the lowest level of education with 54.2% having elementary education or lower, while the rates in Kinh and Tay ethnics were 38.1% and 25.0%, respectively (*p*=0.002). Most of the respondents had a spouse (84.4%) and were never smoking (75.7%). The rate of ever-smoking people in Kinh people was the highest at 82.9%, following by Tay (77.3%) and others (61.7%) (*p*=0.039). The mean annual household income was 489.5 USD (SD = 575.5), of which Tay people had a significantly higher income compared to the other two groups (*p*=0.003).

Table 2 shows that 95% of participants had at least one acute symptom within the last four weeks, and 73.8% had at least one chronic disease in the previous three months. The most common acute symptoms were headache (72.9%), following by back pain (66.4%), cough/sore throat (55.5%), sneezing/runny nose (35.8%), and fever (28.4%). The most common chronic diseases were spine/bone pain (50.2%), gastrointestinal disease (25.6%), and hypertension (23.4%). Kinh people had a significantly higher rate of acute symptoms (e.g., headache, cough/sore throat, and fever) and chronic conditions (e.g., spine/bone pain) compared to people in Tay and other ethnicities (*p* < 0.05).

Table 3 shows that nearly two-thirds of participants used health services in the previous 12 months (63.6%). No difference was found regarding service utilization among different ethnic groups. There were 24.9% of respondents using inpatient service in the last 12 months (mean number of times using service = 2.2, SD = 2.8, *p*=0.969), while there were 47.9% of samples using outpatient services in the previous 12 months (mean number of times using service = 4.8, SD = 4.6). People in other ethnic groups had the lowest number of times using outpatient services (*p*=0.008), while no difference was found among groups regarding the number of times using inpatient services. The number of outpatient medical services used by the Tay participants was higher than that of the Kinh and other ethnic groups (*p* < 0.05). The most commonly used health facilities in the previous year were provincial hospitals (62.6%) and commune health stations (53.2%), and commune health center was the first health facility visited when having an illness of the major samples (72.9%). There was no difference regarding these characteristics among ethnic groups (*p* > 0.05). The average distance from home to the commune health station was 3.6 ± 12.8 km, of which Kinh people had a shorter distance compared to ethnic minorities (*p* < 0.001).

Results of multivariate regression models and their goodness-of-fit indices are presented in Table 4. Compared to Kinh people, Tay people had a higher number of outpatient service use (Coef. = 0.25, *p*=0.04), while people in other ethnics had a lower number of service use (Coef. = -0.64, *p*=0.01). Meanwhile, no difference was found among groups regarding the number of inpatient service use (*p* > 0.05).

Regarding other characteristics, male respondents had a lower number of inpatient service use compared to female ones (Coef. = −1.18, *p* < 0.001). Having secondary, high education, or above reduced the number of inpatient services used in comparison with having elementary education or below (*p* < 0.05). Having a spouse (Coef. = −1.01, *p* < 0.001) or ever smoking (Coef. = −1.79, *p* < 0.001) was associated with a lower number of inpatient service use compared to single or never smoking, respectively. However, people having a spouse (Coef. = 0.24, *p*=0.046) or ever smoking (Coef. = 0.31, *p*=0.03) had a significantly higher number of outpatient service use compared to their counterparts. A higher number of chronic diseases were associated with a higher number of outpatient service use (Coef. = 0.16, *p* < 0.001).

## 4. Discussion

Our study results showed that people in the mountainous areas in the mountainous setting had a high rate of health services used, in which people mainly used outpatient healthcare services. Also, the findings showed that there were ethnic differences in outpatient use and the distance from households to commune health stations.

Our study showed that the majority of participants used healthcare services in the previous 12 months (63.4%), of which the rate of outpatient and inpatient use was 48.2% and 24.4%, respectively. These rates were higher than the previous reports on the general population in Vietnam (36.0%–37.1% for outpatient services and 7%–8.1% for inpatient services) [16, 17]. The rate in this study was also higher than that of another prior study in a mountainous region in 1998 (30%) [1]. Differences among studies could be explained by different demographic characteristics and health status of the study samples. Compared to the previous study in the mountainous areas, the prevalence of health problems in our study was higher, which might partly explain the higher rate of medical service use in our study. Besides, results implied higher healthcare needs among people in mountainous settings than other regions and the need to design prevention programs to reduce morbidity among mountainous people in Bac Kan.

Study results showed that the average distance from home to the commune health station among Kinh people was 3.2 ± 5.5 km, significantly lower than the Tay and other ethnic groups with 3.5 ± 15.3 km and 4.6 ± 3.3 km, respectively. According to a 2016 study, there are ethnic minorities in another mountainous province of Vietnam living 72 km from the nearest health facility [18]. The distance from people's residence to the commune health station in this study was not too far, which perhaps reflects the results of efforts covering the grassroots health system at the locality. However, there were still 15% of the samples who had difficulty in going to the commune health station for medical examination and treatment, and there was no difference among ethnic groups. This showed that although the government had many policies to support and develop grassroots healthcare in mountainous areas [14], distance and geographical issues remain major barriers to access to primary healthcare in this area. This can be a major challenge to ensuring the health of the population, especially given the increasing incidence of noncommunicable diseases that require local and household health management [19]. Developing a village health workers' team can be seen as a solution to the difficulty of accessing such services [20–23].

Multivariate regression results show that compared to the Kinh samples, while Tay people had a higher number of outpatient service use, people in other ethnic groups had a lower number of service use. This result was different from that of previous findings. A study on the use of public health services in a mountainous area of Vietnam showed that ethnic minorities were less likely to use health services than Kinh ethnic group—the major ethnic group in Vietnam [1]. According to UNICEF Vietnam, ethnic minority mothers also use healthcare services less than Kinh mothers due to barriers in distance, costs, language, beliefs, and knowledge [24]. Several reasons can explain these results. Firstly, the Tay people in this study had a higher income than the Kinh individuals; thus, they had sufficient economic conditions to access medical services for their healthcare. Also, we assumed that Kinh people tended to be more likely to have self-treatment habits than ethnic minorities. A study in the highlands of Vietnam showed that Kinh people had a higher likelihood of self-treatment when they were sick [25]. Therefore, the rate and frequency of health service use among Kinh people were lower than those of ethnic minorities. Besides, the research results show that Kinh people have higher education levels than those of other ethnic groups. According to a previous study done in urban areas of Vietnam, high education was a factor promoting self-treatment [26]. These results were consistent with what we observed when the Kinh people participating in the study mainly migrated from lowland areas so that they might have such self-treatment habits from their original place of residence.

We also found several other factors associated with health service use, including gender, education level, marital status, smoking status, and the number of chronic illnesses acquired. Men had a lower number of inpatient use than women, which could be explained by the fact that men had better physical health than women. However, we believed that, due to difficult economic conditions and traditional beliefs, men needed to work hard to support their families. Inpatient treatment could reduce their working time, leading to reduced income. This argument is consistent with the results that married people had lower inpatient use, but higher outpatient use [1, 11]. The results also showed that high educational attainment was related to a reduction in the number of inpatient services used. This is in line with our discussion above, which showed that high levels of education were associated with a higher possibility of self-treatment [26]. In addition, educational attainment was related to health literacy, which was a predictive factor in promoting protective behaviors [27], thereby reducing the severity of health problems [28]. Finally, smokers have a higher number of outpatient service uses than those who never smoke. This was similar to previous studies showing that tobacco smoking was an important factor affecting the onset of diseases and the use of medical services [29, 30]. We have found that smoking reduces inpatient use, which might be because the majority of smokers were men.

This study has several limitations. First, the limitations of a cross-sectional study hinder the ability to conclude causal relationships. Second, although our sample included different ethnic groups because this study was conducted in a mountainous province with a small sample size, it might not be representative of entire ethnic populations in the mountainous region of Vietnam. Therefore, there is a need to conduct further research to expand the sample size and in other provinces. Finally, information about health status, health service use, and access was self-reported, which was more likely to be affected by recall errors or biases.

## 5. Conclusions

This study shows the ethnic differences in outpatient use of health services among communities living in the northern mountains of Vietnam.

## Figures and Tables

**Table 1:** Demographic characteristics by ethnic groups.

Characteristics	Kinh		Tay		Others		Total		*p*
	*n*	%	*n*	%	*n*	%	*n*	%	
Total	42	13.1	230	71.7	49	15.3	321	100.0	

*Gender (n* *=* *321)*									
Female	10	23.8	72	31.3	23	46.9	105	32.7	0.044^*∗*^
Male	32	76.2	158	68.7	26.	53.1	216	67.3	
*Education* (*n* = 318)									
Elementary or lower	16	38.1	57	25.0	26	54.2	99	31.1	0.002^*∗*^
Secondary	17	40.5	119	52.2	15	31.3	151	47.5	
≥ high	9	21.4	52	22.8	7	14.6	68	21.4	

*Marital status (n* *=* *320)*									
Single	7	16.7	35	15.3	8	16.3	50	15.6	0.964^*∗*^
Having spouse	35	83.3	194	84.7	41	83.7	270	84.4	
*Smoking* (*n* = 317)									
Never	34	82.9	177	77.3	29	61.7	240	75.7	0.039^*∗*^
Ever smoking	7	17.1	52	22.7	18	38.3	77	24.3	

	Mean	SD	Mean	SD	Mean	SD	Mean	SD	*p*

Age (*n* = 311)	55.0	14.6	52.0	14.5	47.5	15.7	51.7	14.8	0.041^*∗∗*^
Annual household income (USD)	387.9	352.6	539.0	627.0	368.4	462.9	489.5	575.5	0.003^*∗∗*^

^*∗*^Chi-squared test. ^*∗∗*^Kruskal–Wallis test.

**Table 2:** Health characteristics by ethnic groups.

Characteristics	Kinh		Tay		Others		Total		*p*
	*n*	%	*n*	%	*n*	%	*n*	%	
*Number of acute symptoms (last 4 weeks) (n* *=* *321)*									
None	1	2.4	10	4.4	5	10.2	16	5.0	0.025^*∗*^
One symptom	5	11.9	29	12.6	13	26.5	47	14.6	
Two symptoms or more	36	85.7	191	83.0	31	63.3	258	80.4	

*Acute symptoms (last 4 weeks) (n* *=* *321)*									
Headache	34	81.0	171	74.4	29	59.2	234	72.9	0.043^*∗*^
Backache	31	73.8	150	65.2	32	65.3	213	66.4	0.548^*∗*^
Allergy	7	16.7	46	20.0	4	8.2	57	17.8	0.141^*∗*^
Constipation	4	9.5	40	17.4	9	18.4	53	16.5	0.419^*∗*^
Cough, sore throat	28	66.7	134	58.3	16	32.7	178	55.5	0.001^*∗*^
Sneezing, runny nose	17	40.5	85	37.0	13	26.5	115	35.8	0.307^*∗*^
Fever	20	47.6	63	27.4	8	16.3	91	28.4	0.004^*∗*^
Diarrhoea	2	4.8	21	9.1	4	8.2	27	8.4	0.643^*∗*^
Eyesore	10	23.8	51	22.2	4	8.2	65	20.3	0.071^*∗*^

*Number of chronic diseases (last 3 months) (n* *=* *321)*									
None	4	9.5	59	25.7	21	42.9	84	26.2	0.003^*∗*^
One disease	22	52.4	74	32.2	14	28.6	110	34.3	
Two diseases or more	16	38.1	97	42.2	14	28.6	127	39.6	
*Chronic diseases (last 3 months) (n* *=* *321)*									
Hypertension	10	23.8	57	24.8	8	16.3	75	23.4	0.445^*∗*^
Low blood pressure	8	19.1	40	17.4	6	12.2	54	16.8	0.626^*∗*^
Heart disease	7	16.7	29	12.6	4	8.2	40	12.5	0.469^*∗*^
Gastrointestinal disease	13	31.0	58	25.2	11	22.5	82	25.6	0.636^*∗*^
Spine/bone pain	25	59.5	120	52.2	16	32.7	161	50.2	0.020^*∗*^

^*∗*^Chi-squared test.

**Table 3:** Characteristics of use and access to health services.

	Kinh		Tay		Others		Total		*p*
	*n*	%	*n*	%	*n*	%	*n*	%	
Health service utilization in the last 12 months (*n* = 321)	30	71.4	149	64.8	25	51.0	204	63.6	0.100^*∗*^
*Inpatient service use in the last 12 months (n* *=* *321)*									
None	28	66.7	175	76.1	38	77.6	241	75.1	0.736^*∗*^
One time	8	19.1	32	13.9	7	14.3	47	14.6	
Two times or more	6	14.3	23	10.0	4	8.2	33	10.3	
*Outpatient service use in the last 12 months (n* *=* *321)*									
None	21	50.0	114	49.6	32	65.3	167	52.0	0.183^*∗*^
Once	2	4.8	22	9.6	5	10.2	29	9.0	
Twice or more	19	45.2	94	40.8	12	24.5	125	38.9	
*Health facilities visited in the last 12 months (n* *=* *321)*									
Province hospital	19	54.3	123	63.4	24	66.7	166	62.6	0.511^*∗*^
Commune health center	20	57.1	105	54.1	16	44.4	141	53.2	0.498^*∗*^
Others	5	11.9	28	12.2	3	6.1	36	11.2	0.470^*∗*^
*The first health facility visited when having illness (n* *=* *321)*									
Commune health center	33	78.6	166	72.2	35	71.4	234	72.9	0.671^*∗*^
Others	9	21.4	64	27.8	14	28.6	87	27.1	
Reasons for the selection (*n* = 321)									
Convenient (near home)	26	61.9	146	63.5	34	69.4	206	64.2	0.697^*∗*^
Health insurance registration	27	64.3	156	67.8	28	57.1	211	65.7	0.351^*∗*^
High-quality service	6	14.3	72	31.3	3	6.1	81	25.2	<0.001^*∗*^
Others	7	16.7	64	27.8	2	4.1	73	22.7	0.001^*∗*^
Difficulty when going to the commune health center (*n* = 321)	8	20.0	30	14.2	7	14.6	45	15.0	0.634^*∗*^

	Mean	SD	Mean	SD	Mean	SD	Mean	SD	*p*

*Number of health service utilization*									
Inpatient (*n* = 80)	1.7	1.1	2.3	3.3	1.7	1.2	2.2	2.8	0.969^*∗∗*^
Outpatient (*n* = 156)	4.4	3.3	5.3	4.9	2.2	1.4	4.8	4.6	0.008^*∗∗*^
Distance from home to the commune health center (km)	3.1	5.3	3.4	14.9	4.8	3.2	3.6	12.8	<0.001^*∗∗*^

^*∗*^Chi-squared test. ^*∗∗*^Kruskal–Wallis test.

**Table 4:** Factors associated with health service use.

	Number of inpatient service use				Number of outpatient service use			
	Coef.	*p*	95% CI		Coef.	*p*	95% CI	
*Ethnic*								
Kinh	Ref				Ref			
Tay	0.11	0.75	−0.55	0.77	0.25	0.04	0.01	0.49
Others	-0.09	0.83	−0.94	0.75	−0.64	0.01	−1.13	−0.16
Age	-0.01	0.15	−0.03	0.005	0.003	0.38	−0.003	0.01

*Gender*								
Female	Ref				Ref			
Male	−1.18	<0.001	−1.69	-0.66	0.22	0.10	−0.04	0.48

*Education*								
Elementary or below								
Secondary	−0.42	0.045	−0.84	−0.01	−0.09	0.35	−0.29	0.10
≥ high	−1.15	<0.001	−1.88	−0.42	0.05	0.67	−0.19	0.30

*Marital status*								
Single	Ref				Ref			
Having spouse	−1.01	<0.001	−1.51	−0.50	0.24	0.046	0.004	0.47

*Smoking*								
Never	Ref				Ref			
Ever smoking	−1.79	<0.001	−2.74	-0.83	0.31	0.03	0.04	0.59
Number of acute symptoms (last 4 weeks)	−0.04	0.50	−0.15	0.07	−0.02	0.48	−0.07	0.03
Number of chronic diseases (last 3 months)	−0.09	0.39	−0.30	0.12	0.16	<0.001	0.07	0.25
Distance to the commune health center (km)	−0.02	0.44	−0.07	0.03	−0.03	0.08	−0.06	0.003

Log likelihood	−291.8742	−633.1353						
Prob > chi2	<0.01	<0.01						
AIC	629.7483	1312.271						
BIC	715.0881	1397.61						

## Data Availability

Requests for access to individual subject data may be made to Tam T. Ngo (ngothitam.tlu@gmail.com).
